# Prevalence and associated factors for stunting among 6–12 years old school age children from rural community of Humbo district, Southern Ethiopia

**DOI:** 10.1186/s12889-018-5561-z

**Published:** 2018-05-24

**Authors:** Tesfahun Yonas Bogale, Elazar Tadesse Bala, Minyahil Tadesse, Benedict Oppong Asamoah

**Affiliations:** 1 Health Sciences and Medicine College, Wolaita Sodo University, Wolaita Sodo, Ethiopia; 2 International Maternal and Child Health (IMCH), Department of Women’s and Children’s Health, SE-751 85 Uppsala University, Uppsala, Sweden; 3 Social Medicine and Global Health, Department of Clinical Sciences, Lund University, Malmo, Sweden

**Keywords:** School age children, Stunting, Southern Ethiopia

## Abstract

**Background:**

Stunting is one of the most serious and challenging public health problems in Ethiopia, which constitute a significant obstacle to achieving better child health outcomes. This study aimed to assess the prevalence and factors associated with stunting among 6–12 years old children in Humbo district, Southern Ethiopia.

**Methods:**

This was a cross-sectional study conducted among 633 children 6–12 years old living in Humbo district, Southern Ethiopia, from March to April, 2015. A multistage cluster sampling technique was used to select participants from households in eight Villages in the study area. Height was measured using standard methods and height for age Z-score was computed to assess stunting. EPI info version 3.5.4 was used for data entry, whereas Anthroplus software and SPSS version 20.0 were used for computation of height for age Z-scores and statistical analyses respectively. Simple and multiple logistic regression analyses were used to examine factors associated with stunting in the study sample, using 95% confidence limits (statistical significance set at *p* < 0.050).

**Results:**

Prevalence of stunting was 57%, about, 3.5% were severely stunted, 27.3% moderately stunted and 26.4% mildly stunted, and the mean (SD) was − 1.1 (±1.2). About 7 (1.1%) boys and 15 (2.4%) girls were severely stunted. Age groups 10–12 years had significantly higher rate of stunting than others. Age (AOR = 1.7, 95% CI = 1.1–2.6), big family size (AOR = 4.6, 95% CI = 2.2–9.5) and field disposal of wastes (AOR = 2.7, 95% CI = 1.2–5.8) were factors significantly associated with stunting.

**Conclusion:**

This study exposed high rate of stunting among school age children. Stunting remains a noticeable attribute of rural school age children. Findings suggest the need to implement evidence-based school-aged rural children nutrition policy and strategies as well as need for intervention to improve domestic waste management system in the rural community.

## Background

Child growth is internationally recognized as an important indicator of nutritional status and health in populations. Stunting is one of three anthropometric indices commonly used as an indicator for child growth [1]. Stunted linear growth has become the main indicator of childhood under-nutrition, because it is highly prevalent and has important consequences for health and development [2]. Stunting is defined as height for age Z (HAZ) scores below minus two Z-score of the World Health Organization (WHO) growth reference standard [3]. Height for age Z-score reflects linear growth achieved pre- and post-natal, and its deficits indicate long-term, cumulative effects of inadequacies of health, diet, or/and care. It is associated with higher morbidity and mortality, delayed mental development, poor educational achievement and reduced intellectual capacity, and is a strong predictor of human capital and social progress [4–7].

Stunting is one of the most serious and still one of the challenging public health problems in the world. It is largely invisible in many countries, but affects 165 million children worldwide, 90% of whom live in Africa and Asia [8, 9], making it a major source of concern in developing countries. This makes it a problem of greater magnitude than underweight or wasting. The prevalence of stunting, which is associated with multiple determinant factors intermingled with the long-term lack of proper nutrition, is a significant problem among the people living in resource poor countries. According to WHO, the estimated prevalence of stunting among school-age children aged 5–18 years of age in Africa in 2015 was 37% as compared to the next highest prevalence rate of 23% in Asia [6].

High rates of under-nutrition in Ethiopia constitute a significant obstacle to achieving better child health outcomes. Stunting among children is a chronic problem in developing countries like Ethiopia [10]. Based on researches done in different regions of Ethiopia, the prevalence of stunting among school-age children aged 7–15 years of age varies widely from 9.8 to 48.1% [11]. Research shows that the developmental damage caused by stunting can result in lower intelligence quotient (IQ), poorer educational performance and school completion rates, and a diminished earning capacity. At a macroeconomic level, chronic malnutrition early in life can cost countries up to 11% of their gross domestic product in terms of lower wages and lost productivity [12]. The figures given above show the extent to which the country’s potential work force is faced with growth retardation.

Stunting among children is one of the chronic problems in Ethiopia. Stunting is widely believed to occur mainly in early childhood (mostly by 3 years of age), and through a cumulative process. According to three DHS surveys in Ethiopia the onset of stunting is visible by 6–12 months of age and increases to 24 months of age. In infants < 6 months of age, stunting rates have significantly decreased, going from 22% (2000) and 23% (2005) to 14% in 2011. Stunting rates for children aged 6–24 months went from 49% in 2000, to 47% in 2005, to 35% in 2011. For children under five, rates similarly declined significantly from 54% in 2000, to 49% in 2005, and to 41% in 2011 [13].

The DHS 2011 data revealed that stunting rates are over 40% in Afar, Amhara, Tigray, and Benishangul-Gumu, with the highest rates in Tigray (52%). Rates in Oromia, SNNPR, Dire Dawa, Gambela, Harar and Somali region range from 21 to 32% while Addis Ababa had the lowest rate (13%) [13].

Children stunted at school-age are likely to have been exposed to poor nutrition since early childhood and the degree of stunting tends to increase throughout the school-age years. Limiting the study to children of ages 6–12 years is important because there is a gap in the literature about the nutritional status of this age group. In addition, because girls and boys of this age group have the potential to experience catch-up growth, i.e. growth that will bring them to a normal range of height for age. Even though the problem of child stunting in Ethiopia has been sufficiently recognized, the reasons behind it still need further study.

There is also variation across studies regarding the underlying factors associated with childhood stunting. Besides, to the best of our knowledge, no study has been done to identify factors associated with stunting in the study area. Therefore, this study investigated the important socio-demographic, health and environmental factors associated with stunting in Humbo district.

## Methods

### Study area and setting

This was a cross-sectional study conducted among 6–12 years old children living in 8 villages within Humbo district, located in Southern Nation Nationalities and Peoples Region (SNNPR), which is 360 km from the capital city of Ethiopia. All children within the mentioned age group who lived in the study area at the period of the interviews were eligible to participate in the study.

### Sample size and sampling procedure

The sample size was calculated by based on a formula for estimation of single population proportion. The sample size estimation was based on the following parameters: 95% confidence level, 5% error margin, and 48% prevalence of stunting among school age children 6–12 years old – taken from a previous study in a rural area in Ethiopia [14]. The estimated sample size was then adjusted for a non-response rate of 10% and multiplied by the design effect of 1.5 to obtain a final estimate of 633, the sample size used for this study.

Multistage cluster sampling technique was used for recruitment of study participants. Firstly, eight villages were selected randomly from a total of 39 villages using simple random sampling. The eight selected villages formed the primary sampling units out of which eight clusters (one cluster per village) were selected, also by simple random sampling. The clusters formed the secondary sampling units. From the clusters, households were selected using proportional allocation based on the population size in the given village. One cluster contained 180 households on average. One child was selected from each household. For households which had more than one eligible child within the age group 6–12 years, one child was selected randomly by lottery method. Children with physical deformities of limbs, spine, suffering from diseases and have mental defects were excluded.

### Data collection

The questionnaire used for data collection was adopted from previous studies [14] and pre-tested prior to its use in this study. The questionnaire was first drafted in English language and then translated to Amharic language. Prior to data collection, the purpose of this study was explained to the study participants, their consent to participate was sought and were also informed that their participation in the study was totally voluntary.

The data collectors read out the questions loud and afterwards recorded the responses from the children/caregiver. Age of the child was calculated both from the child’s date of birth and date of interview, since the year of birth is frequently reported incorrectly. In events where birth dates are not recorded or known with certainty, the mother/caregiver was probed for the approximate date of birth based on a local events calendar. Anthropometric measurements were also taken for all children aged 6–12 years to assess their nutritional status.

### Data collectors and measurements

The questionnaires were administered by eight trained and experienced data collectors, who also collected anthropometric information for this study. Two qualified public health officers supervised the process of data collection. The anthropometric measurement taken was height. Standard procedures of height measurements were followed and standardization was done. Height was measured in a standing position using a height meters mounted against a wooden board wall to the nearest of 0.1 cm with detachable sliding head piece which is designed by the United Nations Children’s Fund (UNICEF). Measurements were taken with the children standing barefooted and shoulders erect with their back of heels, buttocks and head touching the wall.

### Data quality control

The questionnaire was pre-tested on comparable setting outside the study area before the actual data collection. The data collectors and supervisors were trained for 2 days on the process of data collection, including how to accurately measure the heights of the children using a measuring scale, according to recommendations for anthropometric measurements. The principal investigator carefully monitored the data collection process.

Quality of the measurements were ensured by first having an expert to measure ten children, followed by the data collectors measuring the heights of the same children twice with a break between the first and second measurements. The average difference between the measurements made by the expert and those of the trained data collectors, and between the first and second measurements of the data collectors were estimated to determine the ‘Technical Error of the Measurements’ (TEM). The relative TEMs for inter and intra examiners for height were 1.8 and 1.7%. Both relative values were above 0.95, the suggested cut-off, indicating a very small error for measurements in the study.

To further minimize systematic error in measurements, each data collector took two repeated measurements and an average height was then calculated and used for the final analyses. In cases where the measures differed or were found to be outside a tolerance limit, the data collector was retrained to carry out the same.

### Data management

Three stastical softwares were used for data management. During the data collection, the data was checked everyday for uniformity and completeness before data entry. The data was first entered into EPi-Info version 3.5.4 statistical software for coding. Afterwards the data was transported into the software Anthroplus, where height to age Z-scores were computed and further checks done to ensure that anomalies resulting from wrongly entered data were corrected. After the initial cleaning, all the z-score values which remained as irregular were cleaned from the file and excluded from further analyses. The cleaned final was then exported to SPSS version 20.0 for further analyses.

### Statistical analyses

Binary (simple and multiple) logistic regression was used to examine the association between stunting and the explanatory variables. From the simple regression models, predictor variables which were associated with the outcome at *p*-value less than 0.25 were selected for inclusion in the multiple logistic regression models. Statistical significance was set at *p* < 0.050 and 95% confidence interval.

### Operational definition

Stunting is defined as height for age Z-scores below minus two (<− 2) Z score of the reference population. Severe stunting is defined as HAZ scores below minus three (<− 3) Z score of the reference population (Table 1).

**Table 1:** Operational definitions of stunting

S.No	Variable	Z – Scores
1.	Stunting	Below minus two (< − 2 Z score) of the reference population
2.	Moderate stunting	Below minus two (< −2 Z score and > or = to − 3 Z score) of the reference population
3.	Severe stunting	Below minus three (<− 3 Z score) of the reference population

## Results

### Socio-demographic characteristics of children and caregivers

The total number of children who participated in the study was 633. Mean (SD) age was 8.9 (±1.9) years and 346 (54.7%) were males. Out of the total households involved about 361 (57%) of the respondents were males and majority were married (571; 90.2%) (Table 1). Two hundred seventy children (42.7%) were from households with eight or more family members. The most common source of drinking water was pipe or shared tap water (384; 60.3%). About 98% (620) of the children were from households that used traditional pit latrine. However only 7 (1.1%) were from households that used ventilated improved pit latrine. Four hundred four children (63.8%) were from households who disposed domestic or household wastes through dumping while 147 (27.5%) disposed their domestic wastes in the field (Table 2).

**Table 2:** Socio-demographic characteristics of (*n* = 633) school-aged children 6–12 years in Humbo district, Southern Ethiopia, March to April 2015

Variables	Result	Frequency (#)	Percent (%)
Sex	Male	346	54.7
	Female	287	45.3
Child’s age in years	6–7	184	29.1
	8–9	194	30.6
	10–12	255	40.3
Sex of caregiver	Male	361	57.0
	Female	272	43.0
Caregiver’s education	No formal education	153	24.4
	Primary education	405	64.0
	Secondary education	69	10.9
	College degree/diploma	6	1.0
Marital status	Married	571	90.2
	Separated	17	2.7
	Widowed/Divorced	45	7.1
Family size	1–4	75	11.8
	5–7	388	61.4
	8+	170	26.8
Source of drinking water	Protected well	71	11.2
	Unprotected well	143	22.6
	River/rain water	34	5.4
	Pipe/tap	382	60.3
Type of toilet facility	Traditional pit latrine	620	97.9
	Ventilated improved	7	1.1
	No facility/bush/field	6	0.9
Disposal site of domestic wastes	Field	147	27.5
	Dumping	404	63.8
	Burning	55	8.7

### Prevalence of stunting

**Fig. 1:**
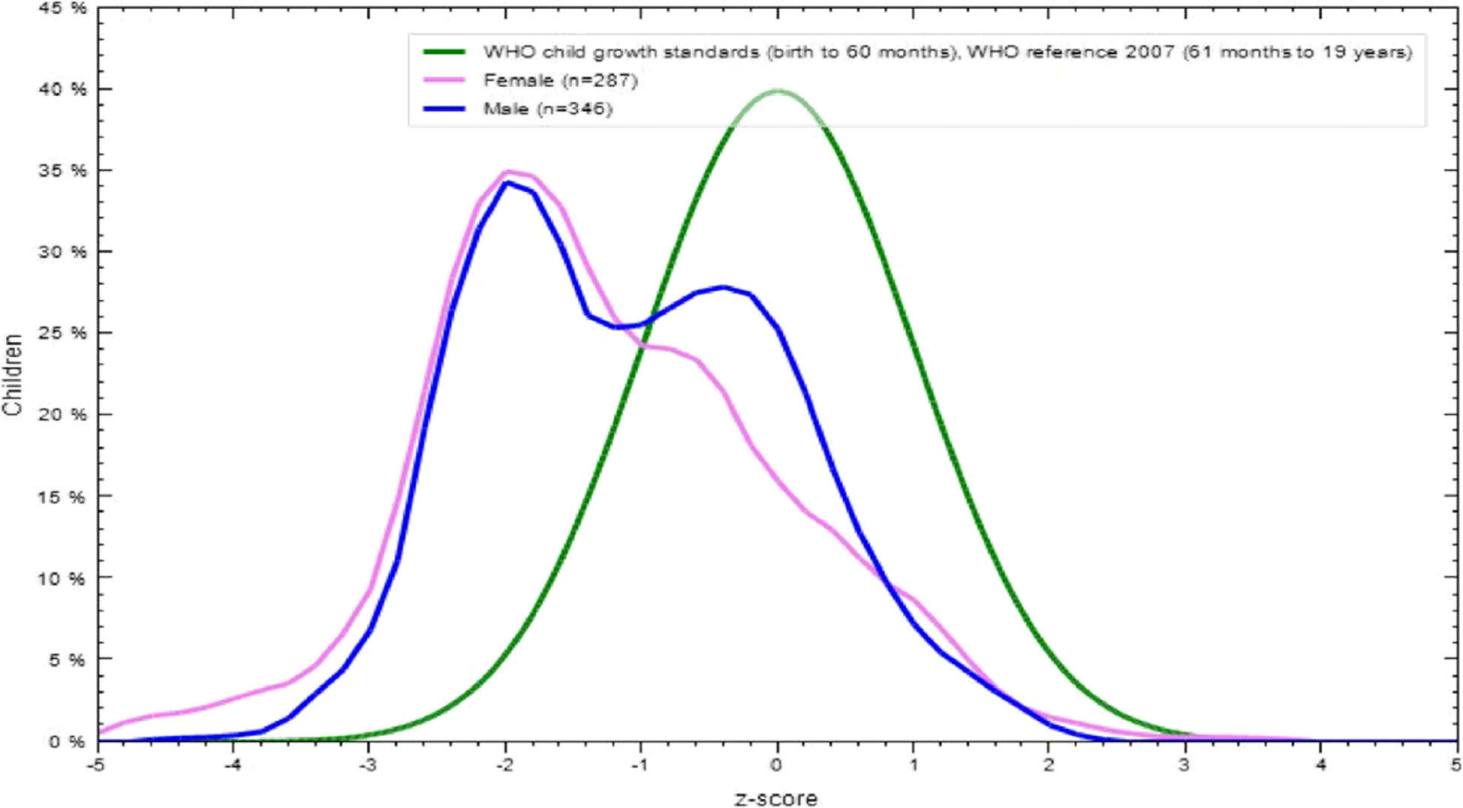
Height-for-age: Z-Score distribution of children as compared to the standard reference population by sex, Humbo district, SNNPR, Ethiopia April 2015

**Fig. 2:**
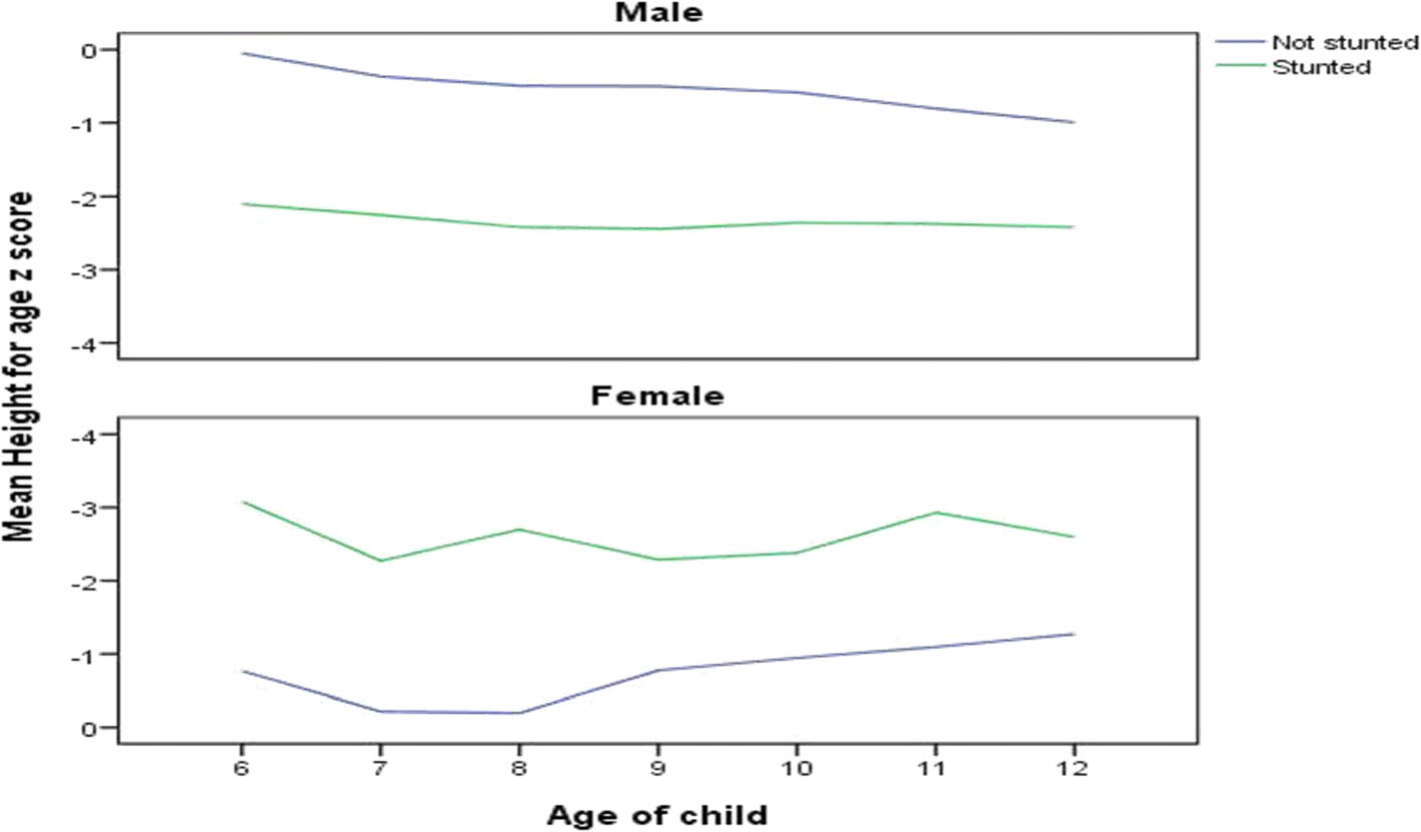
Stunting and mean height for age Z-score by age of children for both sexes in Humbo district, SNNPR, Ethiopia, April 2015

The prevalence of stunting was 57% (95% CI = 50–60%), out of which 23 (3.5%) were severely stunted (< -3SD), 172 (27.3%) moderately stunted (< -2SD) and 167 (26.4%) mildly stunted (< -1SD), and the mean (SD) was − 1.1(±1.2). Seven (1.1%) boys and 15 (2.4%) girls were severely stunted (<-3SD). Figure 1 indicates the height-for-age Z-score distribution of children compared to the standard reference population by sex in the study area (Humbo district). Figure 2 shows the distribution of stunted versus non-stunted children and the respective mean height-for-age Z-score by age and sex.

### Factors associated with stunting

From the bivariate logistic regression analyses, age of child, age and marital status of caregiver, family size and waste disposal area for household wastes were identified as determinants of stunting among school-age children. However, as shown in Table 2, after adjusting for potential confounding variables through multivariate logistic analyses, being between the ages of 10–12 years, having a bigger family size and disposing domestic waste in the field were significantly associated with an increased likelihood of stunting.

Age groups of 10–12 years had 70% increased odds of being stunted when compared to age groups 6–7 years, *p* value < 0.05 (AOR = 1.7, 95% CI = 1.1–2.6). Children from households with large family size (8+) were 4.6 times more likely to be affected by stunting as compared to smaller size families with 1–4 household members, *p* value < 0.05 (AOR = 4.6, 95% CI = 2.2–9.5). Children from the households that dispose domestic wastes in the field were 2.7 times more likely to be affected by stunting compared to those children whose households dispose either by burning or used for biogas or compost, *p* value < 0.05 (AOR = 2.7, 95% CI = 1.2–5.8) (Table 3).

**Table 3:** Bivariate and multivariate logistic regression table for stunting among school aged (*N* = 633) children 6–12 years old in Humbo district, Southern Ethiopia, March to April 2015

Variables	Categories	Stunted		COR (95%CI)	AOR (95%CI)
		N	% (n/N)		
Age of child	6–7	46	7.3	1	1
	8–9	55	8.7	1.2 (0.7–1.9)	1.2 (0.7–1.7)
	10–12	93	14.7	1.6 (1.1–2.6)^*^	1.7 (1.1–2.6) ^*^
Marital status	Married	174	27.5	1	
	Separated	9	1.4	2.6 (0.9–6.8)	
	Widowed/divorced	11	1.7	0.8 (0.4–1.5)	
Caregiver’s age	10–20	2	0.3	1.5 (0.2–8.9)	
	21–30	41	6.5	1.5 (0.6–3.6)	
	31–40	102	16.1	1.7 (0.8–3.9)	
	41–50	40	6.3	1.5 (0.6–3.6)	
	50+	8	1.3	1	
Waste disposal area	Field	62	9.8	2.2 (1.1–4.6) ^*^	2.7 (1.2–5.8) ^*^
	Dumping in to pit	121	19.1	1.7 (0.9–3.4)	1.8 (0.9–3.8)
	Burning/others^**^	11	1.7	1	1
Family size	1–4	10	1.6	1	1
	5–7	169	26.7	2.1 (0.9–4.2)	1.9 (0.9–3.9)
	8+	15	2.4	4.8 (2.4–9.8) ^*^	4.6 (2.2–9.5) ^*^

^*^Key: = p value < 0.05; **=biogas, compost; *COR* crude odds ratio*, AOR* adjusted odds ratio for all the variables in one model

## Discussion

This study revealed the prevalence of stunting to be high (57%) among school-aged children 6–12 years, with severe stunting being twice as high among girls compared to boys. Personal factors such as belonging to the older age group (10–12 years), household and community factors such as family size and poor sanitation (waste disposal in the field) were found to be associated with stunting among the school-aged children.

Generally, the prevalence of stunting observed among school-aged children in this study was higher when compared to other studies conducted in India (9.25%) [15], Nigeria (17.4%) [16], Ghana (50.3%) [17] and Ethiopia (48.1%) [14]. These disparities in findings might be due to differences in study structure, and preventable socioeconomic and political factors such as differential feeding habits, infant and child feeding policies, educational and cultural differences, and other targeted policies rather than differences in their genetic potential to achieve maximum height.

In this study, stunting among boys and girls has no major difference, which is in line with studies conducted in Palestine [18] and Burkina Faso [19], though the prevalence of severe stunting was higher among girls in our study. On the other hand, some previous studies have reported a varied relationship between stunting and gender indicating a higher prevalence among males [5, 20, 21]. This could partly be explained by increased access to food at the grownup age when the females are traditionally involved in the preparation of food, and hence, their better nutritional state compared to the male counterparts. Findings from other studies of a higher prevalence of stunting among females could be due to variances in domestic setups, gender favoritism and parental partialities for male children in that community [22].

It could be seen from our study that as the children grew older, the odds of being stunted increased. Children in the age of 10–12 years had 70% increased odds of being stunted when compared to the youngest children 6–7 years. Similarly previous studies in Ethiopia [14] and Burkina Faso [23] showed the proportion of stunting to be high in the older children. This may be due to prolonged suffering from chronic food shortage. It may also be due to the consequence of eating habits in the community as children are expected to work during the day starved of food whilst growing up [24].

In this study, children belonging to households with large family size (of eight and above members) were more stunted than those belonging to households with small size (1–4 members). Similar findings have been documented in other parts of the world [25–27]. This could be a marker of household poverty, buttressing the fact that stunting is more of a social issue with roots in inequalities across the social strata in the society [28]. A large number of household members could contribute to low levels of childcare and dietary intake [29–31]. There is also the possible risk of overcrowding. This could lead to the spread of diseases, such as chronic respiratory infections and diarrhea which are known to lead to malnutrition.

In addition, our study revealed stunting to be more common in children from households who dispose domestic wastes in the field than those children from households that dispose domestic wastes either through burning or used for biogas/compost. This finding was consistent with multi-country studies conducted in 11 sub Saharan African countries [32], and a national level study in Brazil [33]. With regard to the management of domestic waste, the commonest method observed was that of dumping into a pit, with wastes rarely being collected or receiving any kind of treatment. The management of domestic waste was also found to be unsafe, with trash most commonly being discarded or burned in the field or elsewhere in the village. Considering these inadequate sanitary conditions, it is expected that children may also present with illnesses, due to infectious and parasitic diseases, which tend to worsen nutritional status of children. Given that school-aged children with stunted growth can recover in the early years of their life and evidence from previous research that this recovery is responsive to changes in the household and community environment [34], creates a window of opportunity for targeted anti-poverty policies such as school-feeding programs and public health interventions aimed at improving waste management and reducing environmental contamination for rural areas, poor households and neighborhoods [35, 36].

One of the main strengths of this study is that it is conducted among and children in an age group that has the potential for catch-up growth and therefore could benefit from targeted interventions. The inclusion and assessment of multiple dimensions such as personal, family and community factors that influence stunting is an added strength. However, there are some limitations that need to be considered in this study. Firstly, this study was conducted exclusively in a rural area and therefore lacked the advantage of having to compare with children living in urban settings. Also, the exclusion of children with physical deformities of limbs, spine, suffering from diseases and have mental defects from this study could have resulted to some extent in selection bias, for conditions that are associated with nutritional deficiencies in infants and children.

## Conclusion

This study exposed that stunting rate was high in the study area and all rural school aged children were affected. Children 10–12 years old were more stunted than their younger counterparts. Households with large family size, and children from households that dispose domestic wastes in the field were independently and significantly associated with rising rate of stunting. Since stunting is difficult to treat it calls for preventive measures nested in multiple development sectors.

## Abbreviations

AOR: Adjusted odd ratio
COR: Crude odd ratio
HAZ: Height for age Z score
IQ: Intelligence quotient
OR: Odds ratio
SD: Standard deviation
SNNPR: South Nation Nationalities and Peoples Region
SRS: Simple random sampling
TEM: Technical error of measurements
UNICEF: United Nations Children’s Fund
WHO: World Health Organization
